# Sibelia F. Baker, M. D.

**Published:** 1879-11

**Authors:** 


### DR. SIBELIA F. BAKER.

The following action was taken at the meeting of the Chicago Medical Society, Oct. 20, 1879:

During the summer vacation of this society, death has claimed one of our number, whom we all delighted to honor.

Dr. Sibelia F. Baker was known to all the profession of Chicago as a lady of culture and refinement, a physician of rare ability, one wholly devoted to her profession.

In society and professional matters she was never known to shrink from performing faithfully and efficiently every duty required at her hands. Her many acts of charity to the deserving poor, and her unflinching maintenance of her position where right and wrong were involved, only served to enhance our warmest appreciation of her superior character.

While we are compelled to bow in submission to the stern decree which has removed her from our midst, we cannot abstain from expressing our deep sorrow for our own loss and tendering to her bereaved family our heartfelt sympathy in their great grief.

Let us honor her memory by emulating her virtues.

(Signed,) J. H. ETHERIDGE,<br>EMMA F. GASTON,<br>D. W. GRAHAM, } *Committee.*

Dr. Sibelia F. Baker, the subject of the above tribute, was born in Terry, Ohio, in 1841, and died in Chicago, Ills., Aug. 29th, 1879. Her school days were passed in Zanesville, Ohio, where she remained until failing health, caused by pulmonary disease, bid her seek the more invigorating air of Minnesota.

With returning strength came the resolution to devote her life to the study and practice of medicine. She was graduated from the Woman's Medical College, of Pennsylvania, at Philadelphia, in 1870, and was an honored member of the Alumnæ Association of that institution.

Having had hospital experience in New York and Philadelphia, she located in Coldwater, Michigan, where she practiced successfully four years. Removing to this city she readily gained a high standing in the profession by her skill in, and devotion to, her chosen life-work.

Last year she was honored by the Chicago Medical Society by being nominated as candidate for attending physician to Cook County Hospital. Although the county commissioners did not elect her, yet this expression of professional confidence in her ability could but be gratifying and was fully appreciated by one who richly deserved it.

Dr. Baker was a natural philanthropist, and, in a quiet way, did much to alleviate the condition of the degraded, unfortunate and suffering. She was an earnest worker in the establishment of the Woman's Christian Association Dispensary, of this city, and for two years held the position of medical superintendent in that organization.

Ever zealous in any cause which tended to elevate society, she became an active member of the Illinois Social Science Association, was one of the sanitary committee, and at the time of her death had a paper partially prepared for the annual meeting in October.

To human eyes it seems that she left much other work unfinished, and because the Heavenly Task Master has called her from earthly labor, we mourn the noble woman, the successful physician. EMMA F. G.

---

DEDICATION OF THE NEW LIBRARY HALL OF THE NEW YORK ACADEMY OF MEDICINE. — On the 2nd of October, the members of the medical profession of City of New York had an experience which should interest the members of the profession in Chicago, because it will surely not be many years before Chicago sees a similar sight. The occasion was the dedication of the beautiful new Library Hall of the Academy of Medicine, upon which had been expended the sum of $12,556. Addresses were made by Dr. Fordyce Barker, the president, who presented to the Academy a beautiful marble bust of Mr. T. Spencer Wells, and also by Drs. H. W. Ackland, S. D. Gross, J. S. Billings, Prof. Shattuck, Willard Parker and Austin Flint.

